# Radiation knowledge and perception of referral practice among radiologists and radiographers compared with referring clinicians

**DOI:** 10.1007/s13244-014-0348-y

**Published:** 2014-08-28

**Authors:** Lars Borgen, Erling Stranden

**Affiliations:** 1 Department of Radiology, Drammen Hospital, Dronning gaten 28, 3004 Drammen, Norway; 2 Faculty of Health, Buskerud and Vestfold University College, Grønland 58, 3045 Drammen, Norway

**Keywords:** Radiation protection, Education, Referral and consultation, Questionnaire, Diagnostic imaging

## Abstract

**Objectives:**

To explore if the perception of radiologists and radiographers on referral practice differs from that of referring clinicians, and to see if knowledge of radiation issues and referral guidelines differ between these groups.

**Methods:**

A questionnaire was handed out to 46 radiologists and 36 radiographers in Norway. Findings were compared to corresponding results from a similar already published study on clinicians. Questions dealt with referrals unlikely to affect treatment as well as respondents’ radiation and referral guideline knowledge.

**Results:**

Radiographers estimated the highest proportion of referrals most unlikely to affect treatment (median 20 %) in comparison to radiologists (10 %) and clinicians (5 %). Lack of time, compensating for limited clinical examination and patient expectations were rated as more important reasons for such referrals by radiologists than by clinicians. Radiologists and radiographers possessed significantly better radiation knowledge than clinicians, and were more familiar with referral guidelines.

**Conclusions:**

The perception of radiologists and radiographers differs from that of clinicians, concerning the use of imaging most unlikely to affect treatment, and the reasons for such referrals. Radiologists and radiographers possess better radiation knowledge than referring clinicians, but all groups have a potential in improving their radiation protection knowledge.

***Main Messages*:**

• *Radiographers estimated the highest proportion of referrals most unlikely to affect treatment*.

• *Radiologists rated “getting the patient discharged” as an important reason for such referrals.*

• *Radiologists and radiographers possess significantly better radiation knowledge than clinicians.*

## Introduction

A rapid increase in the volume of medical imaging in recent decades has raised medical, health economic and radiation protection concerns. Imaging overuse and significant volumes of unjustified imaging have been described by several authors [1, 2]. Hence, radiation protection and strengthening of the justification process are important issues of today’s radiology [3, 4].

When exploring unjustified imaging and designing efforts to improve the justification process, a relevant question would be if radiologists and radiographers on one side, and clinicians on the other side, have the same understanding of today’s referral practice. To what extent are today’s practices considered justified on “each side of the table”, and are radiation doses weighted equally?

Good clinical practice relies on adequate imaging reports, which in turn is based on diagnostically acceptable images. Performing relevant image acquisitions depends on sufficient referral information. The radiographer needs to know what to visualise and the radiologist what to look for. In this respective, a lack of mutual understanding on clinical indications and motivations for referring could threaten the quality of radiology and clinical practice.

Several articles have detected scarce radiation knowledge and unawareness of referral guidelines among clinicians [5–9]. These facts underpin the important role of radiologists and radiographers as gatekeepers in the justification process. Hence, radiation and, to some extent, referral guideline knowledge of radiologists and radiographers needs to be sufficient.

Accordingly, the purpose of this study was to see if the perception of radiologists and radiographers on referral practice differs from that of referring clinicians. We also wanted to explore the radiation and referral guideline knowledge of radiologists and radiographers, and to compare this to formerly published findings on referral clinicians.

## Material and methods

In the present study, radiologists and radiographers filled in an anonymous questionnaire. The study did not require approval from a research ethics committee. In a previous paper, results from a corresponding questionnaire handed out to referring clinicians were reported [6].

Our questionnaire was based on literature review, a pilot study of six respondents and individual interviews with four respondents to test face validity. The questionnaire was handed out to respondents during internal meetings at three different hospitals, at national, regional and local levels respectively. All respondents attending the actual meetings were asked to fill in the questionnaire, which took about 15 min. They were not informed about the questionnaire session in advance. The first author supervised these sessions to ensure unaided answers.

The questions concerned, in this order: respondents’ age and gender, their weighting of (six-point scale) radiation dose and four other factors when patients are being referred for imaging, whether they knew of (yes/no) and had used (yes/no) referral guidelines [10], if they received referrals for imaging (radiologists and radiographers)/referred for imaging (clinicians) that most unlikely would affect treatment (yes/no), their approximate rate of such referrals (1, 5, 10, 20 or 50 %) and their rated importance of six listed reasons for such referrals (four-point scale).

The questions exploring referral practice were phrased slightly differently for radiologists and radiographers compared with clinicians [6]. For instance, radiologists and radiographers were asked “Why do you think patients are referred to your department for imaging, when imaging is most unlikely to affect treatment?”, while clinicians were asked “What are your reasons for referring when imaging is most unlikely to affect treatment?”. Also, there were some variations in demographic questions. Except these differences, the questionnaire was organised and phrased identically for all respondent groups.

We also asked about the effective dose (in equivalent number of chest X-rays) from 12 imaging procedures, including radiography, fluoroscopy, multislice computed tomography (MSCT), magnetic resonance imaging (MRI) and ultrasound. Respondents then had to rank the contribution of medical imaging to the mean effective dose for a Norwegian, compared with that of radon in homes, background gamma radiation, pollution from Sellafield in England and food pollution from the Chernobyl nuclear plant accident. Finally, we explored respondents’ knowledge of deterministic and stochastic radiation effects. Except demographic questions, the total number of questions was ten (please see “Appendix”).

A total radiation knowledge score was constructed, ranging from 0 to 71. More detailed information on the questionnaire and the score system can be found in the earlier publication by Borgen et al. [6].

For categorical data, Kruskal-Wallis, Mann–Whitney *U* and chi-squared tests were used as appropriate (see “Results”). Multiple linear regression analysis was used to examine factors that could influence radiation knowledge. Data were analysed using SPSS (version 16; SPSS, Chicago, IL, USA). A two-tailed *p* < 0.05 was accepted as statistically significant.

## Results

All invited radiologists (*n* = 46), radiographers (*n* = 36) participated in the study. In the previous study [6], 213 clinicians participated. The mean age of radiographers was 39.7 years (SD 9.6), radiologists 43.8 years (SD 9.8) and clinicians 44.8 (SD 9.9) years. Female percentage was higher among radiographers (73.5 %) and radiologists (46.7 %) compared with clinicians (23.5 %). Mean number of years of working experience for radiologists was 12.2 (SD 9.6) and for radiographers 12.3 (SD8.9). Corresponding data for clinicians was not obtained.

### Perception of referral practice

We found that 93.5 % of the radiologists and 91.7 % of the radiographers received referrals to imaging most unlikely to affect treatment, while 77.0 % of the referral clinicians admitted such referrals in their own practice (*p* = 0.008, chi-squared test). Radiographers estimated the highest proportion of such referrals (median 20 %) in comparison to radiologists (10 %) and clinicians (5 %) (*p* < 0.001, chi-squared test).

When rating reasons for referrals that were most unlikely to affect treatment (Table 1), “lack of time/getting the patient discharged” was rated more important by radiologists and radiographers than by referring clinicians (*p* < 0.001). The difference was significant between radiographers and clinicians (*p* < 0.001) and between radiologist and clinicians (*p* < 0.001). “To compensate for limited clinical examination” was rated more important by radiologists and radiographers compared with referring clinicians (*p* < 0.001). Again, the difference between radiologists and clinicians (*p* < 0.001) and between radiographers and clinicians (*p* < 0.001) was significant. Expectations of patients were rated as more important by radiographers than by the two other groups (*p* < 0.001). Here, there was a significant difference between radiographers and clinicians (*p* < 0.001) and between radiographers and radiologists (*p* = 0.009). The *p * values were calculated by Kruskal Wallis test and post hoc tests with Bonferroni adjusted Mann–Whitney *U* tests.

**Table 1:** Median score (interquartile range) for what radiologists and radiographers consider the most important reasons for patients being referred to examinations most unlikely to affect treatment, and clinicians’ own reasons for referring patients to such imaging

	Patient expectations	Give the patient the feeling of being taken seriously	Lack of time, “get the patient out of the office”, discharge the patient	Expectations of relatives	Compensate for insufficient clinical examination	Normal findings will reassure the patient
Radiologists	2.0 (2.0)	2.0 (1.0)	2.0 (2.0)	3.0 (1.0)	2.0 (2.0)	2.0 (2.0)
Radiographers	1.0 (1.0)	1.0 (1.0)	3.0 (2.0)	2.0 (2.0)	2.0 (2.0)	2.0 (2.0)
Clinicians [6]	3.0 (1.0)	2.0 (1.0)	4.0 (1.0)	3.0 (1.0)	4.0 (1.0)	2.0 (2.0)

1 = very important, 4 = not important

### Radiation knowledge

Radiologists and radiographers possessed significantly better radiation knowledge than clinicians, *p* < 0.001, multiple linear regression analysis (Table 2).

**Table 2:** Total radiation knowledge score by respondent group

	Mean	*n*	Std. deviation
Radiologists	41.1	46	9.2
Radiographers	38.2	36	7.6
Clinicians 6]	30.4	213	8.4

Mean score was significantly different among the three respondent groups: *p* < 0.001, multiple linear regression analysis

### Weighing of radiation dose

**Fig. 1:**
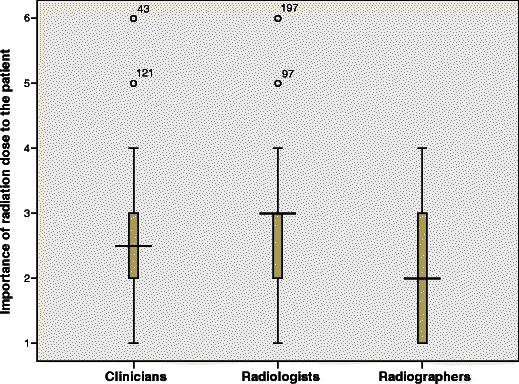
Weighting the importance of radiation dose in relation to referrals; 1 = very important, 6 = not important. Box-and-whisker plot where the *box* represents the interquartile range, the *middle horizontal line* the median and the *whiskers* the range. *Numbered points* are outliers

Radiographers weighted radiation dose as more important than radiologists and clinicians (*p* = 0.03, Kruskal Wallis test) (Fig. 1). Post hoc Mann–Whitney *U* test showed significant difference between radiographs compared with radiologists and clinicians (*p* = 0.012).

### Use of referral guidelines

More radiologists than radiographers and referring clinicians knew of and had used referral guidelines (Table 3). A larger proportion of the radiologists who knew of referral guidelines had used them, compared with corresponding proportions of radiographers and clinicians. Further, 34.8 % of all radiologists, 8.3 % of all radiographers and 2.7 % of all referring clinicians were able to state the website of the Norwegian translation of the European Union referral guidelines (*p* < 0.001, chi-squared tests).

**Table 3:** Number and percentage of respondents who knew and used referral guidelines

	Knew of referral guidelines	Had used referral guidelines
Radiologists	39/46 (84.8 %)	21/46 (45.7 %)
Radiographers	14/36 (38.9 %)	4/36 (11.1 %)
Clinicians 6]	123/212 (58.0 %)	76/213 (35.7 %)

The differences in knowledge and usage were statistically significant, *p* < 0.001 and *p* = 0.003 respectively, both chi-squared tests

## Discussion

### Discussion of findings

Our findings indicate that radiologists and radiographers differ from clinicians in their perception of to what extent patients are referred for imaging that is most unlikely to affect treatment, as well as of the reasons for such referrals. There may be several causes of these discrepancies. The digitalisation of radiology may have increased the psychological and physical distance between the referral clinicians, radiologists and radiographers. Electronic referrals and reports, together with decentralised instant image access have reduced the need for physical meetings. The increasing volume of imaging procedures has put radiologists under pressure [11], marginalising consultations between radiologists and clinicians.

The environment of a radiological department differs substantially from a clinical environment. Radiologists without clinical experience, except basic education and training, are becoming more common [12]. The diversity and complexity of a clinical practice, and hence the need for imaging that at first sight might not seem justified, may be hard to grasp for a radiologist with scarce clinical experience, working with a highly selected patient group. This might also explain some of the differences we find concerning what to emphasise when referring.

Inadequate referrals and failing to communicate sufficient information may also explain some of the differences [13–15]. The quality of referrals varies, and several studies have shown that referrals do not adhere to guidelines [16]. Often, they do not include sufficient clinical information for the radiologist and radiographer to assess the degree of justification [13]. This also might explain some the difference in estimated proportion of referrals most unlikely to affect treatment and reasons for such referrals. Inadequate referrals make it more demanding and time-consuming for the radiologist to prioritise referrals as well as to interpret and report. Hence, the quality of the radiologist’s work may suffer from inadequate referrals.

Radiologists are expected to deliver high volumes of high quality image interpretation. The experience of doing a meaningful job is prerequisite for a durable motivation to deliver what is expected. The radiologists express a relatively high proportion of referrals to imaging most unlikely to affect treatment. If this proportion is perceived as somewhat meaningless, this could indirectly threaten the quality of image interpretation and reports.

One could think that clinicians under-report their proportion of referrals for imaging that is most unlikely to affect treatment, to make their referral practice look adequate and justified. The respondents’ rates were smaller than some reported rates of unjustified imaging (5–10 % vs 20 %) [1, 2, 17]. Our respondents may have underestimated their own rates, but the rate of unjustified and/or treatment-irrelevant imaging in Norway is not known. However, we believe that avoiding person identifiers reduced such bias, and that our most salient findings reflect substantial differences between respondent groups. Non-clinician respondents in our survey may not have had such motivations, as they did not report on their own practice.

Not surprisingly, radiologists and radiographers possess better radiation knowledge than referring clinicians. Education, as well as the nature of everyday tasks, may explain this difference. Better knowledge hopefully makes radiologists and radiographers more able to evaluate the justification of an examination, compared with referring clinicians. However, the potential for improving radiation knowledge is there for all respondent groups. This complies with Lee et al. [7], who found that radiation knowledge was limited among referring clinicians, as well as among radiologists.

Radiographers weighted radiation dose as more important than radiologists and clinicians. This may be explained by the fact that radiographers in their education and daily work are more closely engaged in the physical and radiation-related aspects of radiology.

Referral guidelines are not widely known among clinicians and only about half of clinicians as well as radiologists who knew of such guidelines used them. This may be explained by a lack of marketing of the Norwegian translation of the EU referral guidelines. The translation was only available online and not as a booklet. There was no regular update. Inadequate implementation of referral guidelines have also been described by other authors [18, 19].

This study shows that the considerations of radiologists and radiographers differ from that of the clinicians, concerning the use of imaging most unlikely to affect treatment, and the reasons for such referrals. Radiologists and radiographers possess better radiation knowledge than referring clinicians.

To create a common ground and mutual understanding of justification and referral practice, there seems to be a need for improved communication and closer collaboration between radiologists, radiographers and referring clinicians. A common understanding of justification and radiation protection issues could facilitate a meaningful use of the radiological department, mutual confidence as well as sustained high quality radiology.

### Strengths and limitations

A 100 % participation rate and unprepared, unaided responses to our questionnaire yielded more valid data than achievable in a postal or e-mail survey. However, the use of questionnaires has inherent limitations, as some answers reflect respondents’ subjective opinion. Respondents’ self-reporting on their own practice should be interpreted with care. The proportion of referrals to imaging that most likely will not affect treatment may be higher or lower, if investigated with more objective methods. On the other side, the subjective perception on current referral practice is exactly what we wanted to investigate, and knowledge of the differences we find among the respondent groups could be of value when designing efforts to improve the justification process.

We recruited respondents among radiologists and radiographers at institutions from three different hospital levels at morning meetings where residents, junior doctors and senior doctors attend. Clinicians were recruited at such meetings or courses of general interest, and the age of the respondent groups of clinicians corresponded to the age of these groups on a national level [6, 20]. Age and female percentage among radiologists were 45 years and 44 % on a national level versus 44 years and 47 % in our sample [A. Taraldset, Chief of Statistics, Norwegian Medical Association, pers. commun., 13 May 2014]. For radiographers, corresponding numbers were 44 years and 75 % on a national level and 40 years and 74 % in our sample [A. Petterson, Leader of the Norwegian Society of Radiographers, pers. commun., 13 May 2014]. Thus, we believe that our respondent samples may be fairly comparable to the corresponding groups on a national level.

## Appendix

**Table 4:** Questions about medical radiation use and radiation protection

Questions	Response categories
To what extent should the listed criteria be important when a patient is being referred for imaging?	Weighting of importance 1–6; 1 = very important, 6 = not important
Radiation dose to patient	
Patient’s wish	
Impact on diagnosis	
Impact on treatment	
Impact on patient’s future health	
Do you know of imaging referral guidelines, where referrers can seek information on which investigations are indicated for which conditions?	Yes/No
Have you ever used such referral guidelines?	Yes/No
Are patients referred to your department for imaging in cases when you consider it most unlikely that the imaging results will affect treatment of the patient?	Yes/No
If yes, what is the proportion of such referrals among the referrals to your department (circa)?	1 %, 5 %, 10 %, 20 %, 50 %
Why do you think patients are referred to your department for imaging, when imaging is most unlikely to affect treatment? Please weight the listed reasons	Weighting of importance 1–4; 1 = very important, 4 = not important
Patient expectations	
Give the patient the feeling of being taken seriously	
Lack of time, “get the patient out of the office”, discharge the patient	
Expectations from relatives	
Compensate for insufficient clinical examination	
Normal findings will reassure the patient	
Please estimate the effective dose of the listed imaging procedures, compared to a chest X-ray (front and side projection). Please put a mark, even if you are uncertain^a^	Corresponding numbers of chest X-rays (front and side projection); 0–1, 1–10, 10–50, 50-200
Cerebral CT	
Pelvic radiography	
Cerebral MRI	
Intravenous pyelography	
Chest CT	
Barium meal fluoroscopy	
Barium enema	
Abdominal CT	
Kidney ultrasound	
Thoracic spine radiography	
Sinus X-ray	
Sinus CT	
We ask you to rank the contributors to the mean effective radiation dose for a Norwegian patient in 2006	Rank, 1 = largest contributor, 5 = smallest contributor
Medical imaging	
Radon in homes	
Background gamma radiation	
Pollution from Sellafield in England	
Pollution from the Chernobyl nuclear plant accident	
Detrimental effects of radiation are divided into deterministic and stochastic effects. Are you familiar with these terms? If yes, go to next question	Yes/No
This is a list of potential detrimental effects of radiation. Please mark whether you think these effects are stochastic or deterministic (one mark per effect)	Stochastic/Deterministic
Leukaemia	
Infertility	
Fetus abnormalities	
Genetic adverse effects	
Cataract	
Lung cancer	

^a^Estimates of effective dose were compared with national reference values or—when such values were lacking—with doses measured at the first author’s department
